# Molecular Profiling of Ticks and Associated Pathogens: First Report of *Rickettsia sibirica*, *Rickettsia slovaca*, and *Babesia microti* in Ticks From Pakistan

**DOI:** 10.1155/tbed/3157047

**Published:** 2025-10-06

**Authors:** Muhammad Kashif Obaid, Jin Luo, Shuaiyang Zhao, Zhancheng Tian, Shakir Ullah, Jehan Zeb, Guangyuan Liu, Jianxun Luo, Hong Yin, Muhammad Rashid, Qiaoyun Ren, Guiquan Guan

**Affiliations:** ^1^State Key Laboratory for Animal Disease Control and Prevention, Key Laboratory of Veterinary, Parasitology of Gansu Province, Lanzhou Veterinary Research Institute, Chinese Academy of Agricultural Sciences, Lanzhou, Gansu, China; ^2^Department of Zoology, Abdul Wali Khan University, Mardan, Pakistan; ^3^School of Public Health, The University of Hong Kong, Hong Kong SAR, Hong Kong, China; ^4^Department of Parasitology, Faculty of Veterinary and Animal Sciences, The Islamia University of Bahawalpur, Punjab 63100, Pakistan; ^5^Hebei Key Laboratory of Animal Physiology, Biochemistry and Molecular Biology, Hebei Collaborative Innovation Center for Eco-Environment, Ministry of Education Key Laboratory of Molecular and Cellular Biology, College of Life Sciences, Hebei Normal University, Shijiazhuang 050024, China

**Keywords:** *Babesia microti*, public health, *Rickettsia sibirica*, *Rickettsia slovaca*, zoonotic infection

## Abstract

Global distribution of ticks and their associated tick-borne pathogens (TBPs) presents substantial health concerns for both humans and animals. The present study aimed to investigate the distribution, morpho-molecular identification, and associated TBPs of diverse tick species collected from Khyber Pakhtunkhwa and Punjab provinces of Pakistan. Morphological identifed ticks were molecularly confirmed via cytochrome oxidase I (*COI*) and *16S rRNA* genes, which showed 15 different tick species. Among them, we found the highest prevalence rate of *Rhipicephalus* (*Rh*) *microplus* (404/1803; 22.41%), while the lowest prevalent tick species were *Haemaphysalis* (*Hae*) *montgomeryi* (44/1803; 2.44%). Similarly, the highest tick load was found on cattle (544/186; 2.92), while least was found on goats (272/164; 1.66). Various TBPs, including *Rickettsia* (*R*) spp. (via *gltA*, *sca4*, *ompA*, *ompB* genes), *Anaplasma* (*A*) sp. (via *16S rRNA* gene), *Coxiella burnetii* (via *IS1111* gene), Piroplasm and *Hepatozoon* (*H*) spp. (via *18S rRNA* gene) were screened. The *Rh. microplus* tick species showed highest positivity rate (11.63%) for various TBPs, whereas *Hae. sulcata* ticks were recorded as TBPs free in current study. Among the detected TBPs, *Coxiella burnetii* was most prevalent (1.72%), followed by *A. phagocytophilum* & *R. slovaca* (1.44% each), *R. sibirica* (0.78%), *R. raoultii* (0.61%), *H. canis* (0.55%), *R. conorii* subsp. *raoultii* (0.50%), *Hepatozoon* sp., (0.44%), *Theileria* (*Th*) *uilenbergi* (0.39%), *Babesia* (*B*) *microti* (0.33%), and *Th. luwenshuni* (0.28%). This study provides the first report of TBPs, including *R. slovaca*, *R. sibirica*, and *B. microti*, in ticks from Pakistan. Phylogenetic analysis was performed based on the aforementioned genetic markers, in which ticks and their associated TBPs formed distinct clades with their corresponding isolates. This research work provides a deep insight regarding the characterization of different ticks and their associated TBPs in Pakistan and, significantly, it presents evidence of potential zoonotic threats to both animal and public health due to these newly detected pathogens.

## 1. Introduction

Ticks (Ixodidae and Argasidae), blood-feeding ectoparasites, exist globally due to their adaptability to various environmental conditions from forests to grasslands [1]. The movement as well as survival of these ectoparasites depends on the environmental factors (temperature and humidity) [2]. Beyond their ecological role, ticks serve a crucial role in the transmission of several types of pathogens, posing a significant threat to both human and animal health worldwide [3]. These tick-borne pathogens (TBPs) could be Rickettsiales bacteria (*Rickettsia* spp., *Anaplasma* spp.), *Coxiella burnetii*, Piroplasms (*Babesia* spp. and *Theileria* spp.), and *Hepatozoon* spp. [4, 5].

Among these, spotted fever group (SFG) rickettsiae are emerging as health concerns, with 48 reported species infecting humans, animals, and arthropods [6]. Notably, *Rickettsia* (*R*) *sibirica*, *R. slovaca*, and *R. raoultii* are associated with febrile illnesses and potential complications [7]. *Rickettsia sibirica*, which causes Siberian typhus (North Asian tick typhus), is prevalent in several Asian regions (China, Mongolia, Kazakhstan, and Russia) and is mainly transmitted via *Dermacentor* and *Haemaphysalis* (*Hae*) ticks [8–12]. Additionally, *R. slovaca* was first isolated in 1968 from *Dermacentor* ticks in central Slovakia [13] and declared as human pathogen in 1997 by Raoult et al. [14], which causes scalp eschar and neck lymphadenopathy (SENLAT) syndrome that arise after bite of *Dermacentor* ticks [15]. In 1999, the *R. raoultii* bacterium was initially found in *Dermacentor* and *Rhipicephalus* (Rh) ticks from the former Soviet Union [16, 17], which was further confirmed by using embryo-based cell lines of *Rh. microplus* and *Rh. sanguineus* tick species [18, 19]. In humans, *R. raoultii* has been reported as the causative agent for tick-borne lymphadenopathy (TIBOLA) or *Dermacentor*-borne necrosis erythema and lymphadenopathy (DEBONEL) [12, 20].

Other significant pathogens include various *Anaplasma* (*A*) spp., including *A. phagocytophilum*, *A. centrale*, *A. capra*, *A. platys*, and *A. marginale*, which have been detected in numerous arthropod vectors and host animals globally [21–23]. *Anaplasma phagocytophilum* is a notable zoonotic pathogen causing granulocytic anaplasmosis in humans, domestic ruminants, horses, dogs, and occasionally in cats [24, 25]. Similarly, *Coxiella* (C) *burnetii*, the causative agent of Q-fever/coxielliosis, is mainly transmitted to humans and livestock through inhaling the contaminated aerosols, although ticks, particularly *Rhipicephalus* and *Ixodes* genera, can act as both reservoirs and hosts [26, 27]. In addition, the *Hepatozoon* (H) spp. infect numerous vertebrates, including livestock and wildlife [28], with *H. canis* and *H. felis* species being mainly transmitted by *Rhipicephalus* ticks to dogs, cats, goats, and sheep [29, 30], resulting in substantial morbidity in immunocompromised or coinfected animals [31].

Among Piroplasms (intracellular protozoan parasites), *Babesia* and *Theileria* are the most prominent genera, infecting several vertebrate animals, especially mammals, through the transmission of mainly Ixodid ticks [3]. Various *Theileria* species, such as *Th. luwenshuni* and *Th. uilenbergi*, are reported in small ruminants (sheep and goats) in Asia [32–34], causing severe cases of hemolytic anemia and immune suppression, which results in death [35]. Concurrently, different species of *Babesia* (*B*) specifically *B. microti* is a zoonotic pathogen infecting humans and causing babesiosis in approximately more than 2000 humans annually [36–38] and in ticks across the world [39].

The diverse climatic conditions in Pakistan, including significant variations in temperature, topography, and availability of hosts, create conducive environments for the proliferation and dissemination of ticks and their TBPs [40–43]. Considering the increasing importance of TBPs, there remains substantial inadequacy of information about their vector, genetic diversity, pathogenicity, and zoonotic transmission in Pakistan. Hence, this study was designed aiming at addressing this critical epidemiological gap by providing a thorough molecular characterization and phylogenetic analysis of ticks and TBPs across several geographical localities of Pakistan. These findings are crucial for enhancing our understanding of TBPs landscape in Pakistan and offer essential insights into the imminent threats to the targeted livestock, wildlife, and public health.

## 2. Materials and Methods

### 2.1. Ethical Approval

The current proposed research work received approval from the ethical committee of the Department of Parasitology at Lanzhou Veterinary Research Institute, Chinese Academy of Agricultural Sciences (LVRI, CAAS), China (Permission Number: LVRIAEC-2023−043). During sampling, all animals were handled carefully to minimize the stress in accordance with the “Pakistan's Prevention of Cruelty to Animal Act 1890” and the Animal Ethics procedures and Guidelines of the People's Republic of China.

### 2.2. Study Localities

**Figure 1:**
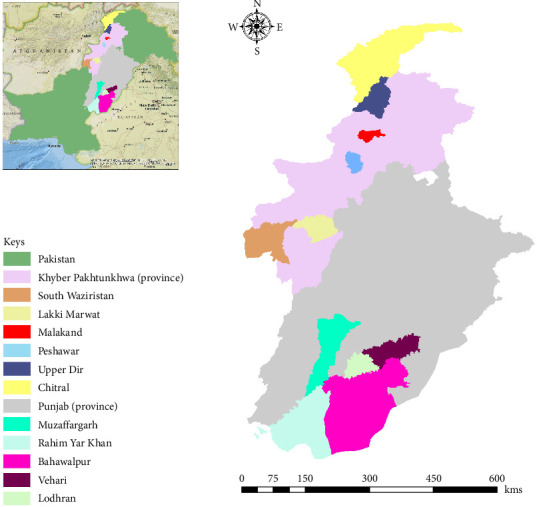
Map presenting current study locations of sampling, designed via ArcGIS 10.3.1 (ESRI, Redlands, CA, USA).

Sampling was carried out across various districts in Khyber Pakhtunkhwa province, including Chitral (35.708078, 71.752235), Upper Dir (35.232782, 72.087904), Peshawar (33.993694, 71.552406), Lakki Marwat (32.609942, 70.866833), South Waziristan (32.257411, 69.626472), and Malakand (34.536296, 71.879816). Additionally, districts from Punjab provinces, namely Bahawalpur (29.379308, 71.687261), Muzaffargarh (30.071242, 71.191487), Lodhran (29.533927, 71.629120), Rahim Yar Khan (28.421022, 70.307435), and Vehari (30.047452, 72.355508). The collected coordinates of each district were processed in Microsoft Excel 2013 (Microsoft 365) to make a study area map via ArcGIS 10.3.1, showing the distribution of ticks in targeted localities (Figure 1).

### 2.3. Sampling Animals and Ticks Collection

A total of 1339 various host animals were examined for tick infestation during March 2024 to October 2024, which included 304 cattle (*Bos taurus*), 210 camels (*Camelus dromedarius*), 284 goats (*Capra hircus*), 182 sheep (*Ovis aries*), 218 dogs (*Canis lupus* familiaris), 102 chickens (*Gallus gallus*), and 39 Lizards (*Varanus bengalensis*). A convenient sampling strategy was employed within each district to ensure extensive representation. Ticks were carefully removed from individual host animals and immediately kept in a separate labelled Eppendorf tube containing 100% ethanol. All samples were transported to the Laboratory of Molecular Parasitology and Immunology, Department of Parasitology, Faculty of Veterinary and Animal Sciences, The Islamia University of Bahawalpur, Pakistan, followed by morphological identification under the stereomicroscope by comparing their distinguished features using standard keys [44–49]. Morphologically identified ticks were cleaned with 70% ethanol and DNase-free water (ddH_2_O), followed by homogenization via a TissueLyser-2 (QIAGEN, Germany). These samples were processed for genomic DNA (gDNA) extraction by a QIAamp DNA Mini Kit (QIAGEN, Germany, LOT-175018422, cat. no. 51,306) according to the manufacturer's instructions. Quantity and purity of extracted gDNA were analyzed via Nanodrop ND-1000 spectrophotometer (Thermo Fisher Scientific, Waltham, MA, USA) and the gDNA with 260/280 (1.6–2.0) as well as concentration of ≥ 50 ng/µL were considered suitable for further processing, that were transported to LVRI, CAAS, China and stored at −20°C until further processing.

### 2.4. Molecular Processing

Extracted gDNA from individual ticks was subjected to conventional PCRs targeting *16S rRNA* and cytochrome oxidase I (*COI)* genes. Subsequently, these ticks were screened for associated TBMs using various primers, including *gltA*, *sca4*, *ompA*, *ompB*, *18S rRNA*, *16S rRNA*, and *ISIIII* genes (Table 1). Each PCR reaction was performed in a 25 µL volume, containing 1 µL (10 µM) of each (forward and reverse) primer, 2 µL of gDNA (template), 8.5 µL of ddH_2_O, and 12.5 µL of 2x Rapid Taq Master*Mix* (Vazyme, China), with their respective cycling conditions (Table 1). In every amplification run, an appropriate positive control and negative control (NTC) were used to confirm the accuracy and integrity of the PCR results. Each NTC were contained of all reaction components except template DNA (replaced by ddH_2_O). All the amplified PCR products were electrophoresed on a 1.5% agarose gel, stained with 4 µL GoldView (cat no. G8140, lot no. 2,017,204), and visualized under the UV light using Gel Doc System (BioDoc-It Imaging Systems UVP, LLC). Each amplicon was purified via Omega Bio-Tek's E.Z.N.A Gel Purification Kit (Lot: D2500020000L28u029, E.Z.N.A. Gel Extraction Kit (V-spin)-Omega Bio-Tek), followed by confirmation using PCR with respective gene primers, and finally sent to the commercial company (Sangon Biotech Co., Ltd., China) for Sanger sequencing.

### 2.5. Data Processing

Additionally, all the obtained sequences were viewed and purified in SeqMan v. 5.00 (DNASTAR) and BioEdit v. 7.0.5 to get the finalized trimmed sequences. All homologous consensus sequences for each gene generated via SeqMan v. 5.00 were submitted to National Center for Biotechnology Information (NCBI) to obtain the respective accession numbers. Phylogenetic analysis was performed by molecular evolutionary genetics analysis (MEGA-X) based on each respective gene [60] via neighbor-joining [61] and maximum likelihood [62] methods along with the application of 1000 bootstrapping replications in different models, including LogDet (Tamura-Kumar) [63], p-distance [64], Kimura 2-parameter model [62] and maximum composite likelihood model [65]. Moreover, all the collected data regarding epidemiology and distribution of ticks were processed in Microsoft Excel 2016 (Microsoft 365) for descriptive statistics and designing the study map. Prevalence and confidence intervals for TBPs were identified using R 4.1.0 [66].

## 3. Results

### 3.1. Overall Distribution of Host Animals and Ticks

A total of 1339 various host animals were examined in the current study, among which 749/1339 (55.94%) were found to be infested with a variety of tick species. Tick infestation was higher in district Peshawar (74/749; 9.88%), followed by South Waziristan (71/749; 9.48%), Malakand (70/749; 9.35%), Lodhran (69/749; 9.21%), Upper Dir and Vehari each (68/749; 9.08%), Chitral (67/749; 8.95%), Lakki Marwat and Rahim Yar Khan each (66/749; 8.81%), and Muzaffargarh and Bahawalpur each (65/749; 8.67%). Specifically, the highest number of cattle were found infested with ticks (186/749;24.83%), followed by Goats (164/749; 21.89%), Camels (118/749; 15.75%), Dogs (116/749; 15.49%), Sheep (100/749; 13.35%), Chickens (47/749; 6.27%), and Lizards (18/749; 2.40%) (Table 2).

Additionally, a total of 1803 ticks were collected, with the highest number of nymphs [N (1071/1803; 59.40%)], followed by female ticks *(F* [617/1803; 34.22%]) and male ticks *(M* (115/1803; 6.38%]). The distribution of overall ticks in different targeted localities were as follows: Peshawar district had the highest number of ticks (180/1803; 9.98%, *N* = 116, *F* = 53, *M* = 11); followed by Chitral (173/1803; 9.60%, *N* = 92, *F* = 67, *M* = 14); Lakki Marwat (171/1803; 9.48%, *N* = 96, *F* = 62, *M* = 13); Upper Dir (169/1803; 9.37%, *N* = 101, *F* = 57, *M* = 11); South Waziristan (168/1803; 9.32%, *N* = 92, *F* = 64, *M* = 12); and Malakand (166/1803; 9.21%, *N* = 93, *F* = 59, *M* = 14) in Khyber Pakhtunkhwa province. Whereas, in the districts of Punjab province, notable tick population were from Vehari (164/1803; 9.09%, *N* = 90, *F* = 62, *M* = 12), Bahawalpur (163/1803; 9.04%, *N* = 101, *F* = 54, *M* = 8), Rahim Yar Khan (151/1803; 8.37%, *N* = 103, *F* = 44, *M* = 4), Muzaffargarh (150/1803; 8.32%, *N* = 95, *F* = 49, *M* = 6), and Lodhran (148/1803; 8.21%, *N* = 92, *F* = 46, *M* = 10) are listed in descending order. The details of morpho-molecularly identified tick species, including *Argas* (*Ar*) *persicus*, *Ixodes* (*Ix*) *kashmiricus*, *Amblyomma* (*Am*) *gervaisi*, *Rh. microplus*, *Rh. turanicus*, *Rh. sanguineus*, *Rh. haemaphysaloides*, *Hyalomma* (*Hy*) *dromedarii*, *Hy. detritum*, *Hy. excavatum*, *Hy. anatolicum*, *Hy. asiaticum*, *Hae. montgomeryi*, *Hae. bispinosa*, and *Hae. sulcata*are described in Table 2.

### 3.2. Host-Wise Distribution of Tick Species

Host-wise overall distribution of ticks presented the highest rates for cattle (544/1803; 30.17%), followed by camels (324/1803; 17.97%), dogs (285/1803; 15.81%), goats (272/1803; 15.09%), sheep (241/1803; 13.37%), chicken (92/1803; 5.10%), and lower distribution was found on lizards (45/1803; 2.49%). Infestation of each individual tick species was recorded on each type of targeted host animals, which present the distribution of individual tick species across each host animal. Specifically, *Ar. persicus*, *Am. gervaisi*, *Hy. excavatum*, *Hy. dromedarii*, and *Hae. montgomeryi* tick species were found exclusively (100%) on chicken, lizards, dogs, camels, and sheep, respectively. Further details regarding the distribution of remaining tick species on their specific host animals. Moreover, overall tick load on all the targeted animals were found 1803/749 (2.41), including the highest tick load on cattle (544/186; 2.92), followed by camels (324/118; 2.75), lizards (45/18; 2.50), dogs (285/116; 2.46), sheep (241/100; 2.41), chicken (92/47; 1.96), and goats (272/164; 1.66) as presented in Table S1.

### 3.3. Prevalence and Distribution Rates of Tick-Borne Microorganisms

The obtained prevalence rate of overall TBM(s) were recorded as 153/1803 (8.48%, CI: 7.27%−9.82%) in the targeted tick species. The TBMs were further categorized into four major groups, including the highest prevalence rate of Rickettsiales bacteria (86/1803; 4.77%, CI: 3.82%−5.91%), followed by *C. burnetii* (31/1803; 1.72%, CI: 1.18%−2.43%), while 18/1803 (0.99%, CI: 0.59%−1.63%) of prevalence rates for each Piroplasms and *Hepatozoon* were recorded across various obtained tick species during current study. The detailed specie-wise prevalence of Rickettsiales was noted as; *A. phagocytophilum* & *R. slovaca* (26/1803; 1.44%, CI: 0.94%−2.09%) each, *R. sibirica* (14/1803; 0.78%, CI: 0.46%−1.30%), *R. raoultii* (11/1803; 0.61%, CI: 0.32%−1.09%), and *R. conorii* subsp. *raoultii* (9/1803; 0.50%, CI: 0.23%−0.96%). Specie-wise prevalent Piroplasms were *Th. uilenbergi* (7/1803; 0.39%, CI: 0.16%−0.79%), followed by *B. microti* (6/1803; 0.33%, CI: 0.12%−0.72%), and *Th. luwenshuni* (5/1803; 0.28%, CI: 0.09%−0.66%). Additionally, *C. burnetii*, *H. canis*, and *Hepatozoon* sp. were recorded with prevalence rates of 31/1803 (1.72%, CI: 1.18%−2.43%), 10/1803 (0.55%, CI: 0.26%−1.04%), and 8/1803 (0.44%, CI: 0.19%−0.87%), respectively (Table 3).

Additionally, tick species belonging to genus *Rhipicephalus* in the current study showed higher positivity rates for various TBMs (81/788; 10.28%, CI: 8.24%−12.68%), while genus *Haemaphysalis* was found with least positive rates (10/203; 4.93%, CI: 2.57%−9.20%). However, the positivity rates for overall TBMs of each individual tick species were found as highest by *Rh. microplus* (47/404; 11.63%, CI: 8.76%−15.19%), followed by *Hy. anatolicum* (16/154; 10.39%, CI: 6.55%−15.93%), *Rh. haemaphysaloides* (15/153; 9.80%, CI: 5.92%−15.34%), *Rh. turanicus* (11/116; 9.48%, CI: 8.76%−15.19%), *Hy. asiaticum* (12/130; 9.23%, CI: 5.17%−15.86%), *Hae. bispinosa* (7/80; 8.75%, CI: 3.86%−17.65%), *Hy. excavatum* (4/56; 7.14%, CI: 2.21%−16.79%), *Ix. kashmiricus* (6/86; 6.98%, CI: 2.87%−14.86%), *Rh. sanguineus* (8/115; 6.95%, CI: 3.06%−13.19%), *Hae. montgomeryi* (3/44; 6.82%, CI: 1.87%−18.06%), *Am. gervaisi* (3/45; 6.67%, CI: 1.83%−17.75%), *Hy. dromedarii* (11/170; 6.47%, CI: 3.55%−11.41%), *Hy. detritum* (5/79; 6.32%, CI: 2.15%−13.88%), and *Ar. persicus* (5/92; 5.43%, CI: 1.83%−11.96%), while *Hae. sulcata* was free from any type of TBMs in this study (Table 3).

Two new Rickettsiales bacteria, including *R. slovaca* and *R. sibirica* were reported for the first time in different tick species from Pakistan. Specifically, the *R. slovaca* were detected in *Ix. kashmiricus* at the highest positivity rate (4/86; 4.65%, CI: 1.53%−11.23%), followed by *Hy. detritum* (3/79; 3.80%, CI: 1.03%−10.43%), *Hy. anatolicum* (5/154; 3.25%, CI: 1.25%−7.42%), *Rh. microplus* (12/404; 2.97%, CI: 1.63%−5.11%), and *Rh. haemaphysaloides* (2/153; 1.31%, CI: 0.28%−4.66%). Moreover, *R. sibirica* was found highly positive in *Hy. dromedarii* (5/170; 2.94%, CI: 1.14%−6.85%) followed by *Rh. turanicus* (3/116; 2.59%, CI: 0.74%−7.23%), *Am. gervaisi* (1/45; 2.22%, CI: 0.11%−12.00%), *Rh. haemaphysaloides* (3/153; 1.96%, CI: 0.56%−5.60%), and *Hy. asiaticum* (2/130; 1.54%, CI: 0.33%−5.46%). In Piroplasms, two *Theileria* species (*Th. luwenshuni* & *Th. uilenbergi*) and one novel *Babesia* (*B. microti*) have been identified from various tick species, including *Rh. microplus*, *Rh*. *haemaphysaloides*, *Hae. bispinosa*, *Hy. anatolicum*, and *Hy. asiaticum* (Table 3).

### 3.4. Sequence Similarities and Phylogenetic Analysis of Tick Species

**Figure 2:**
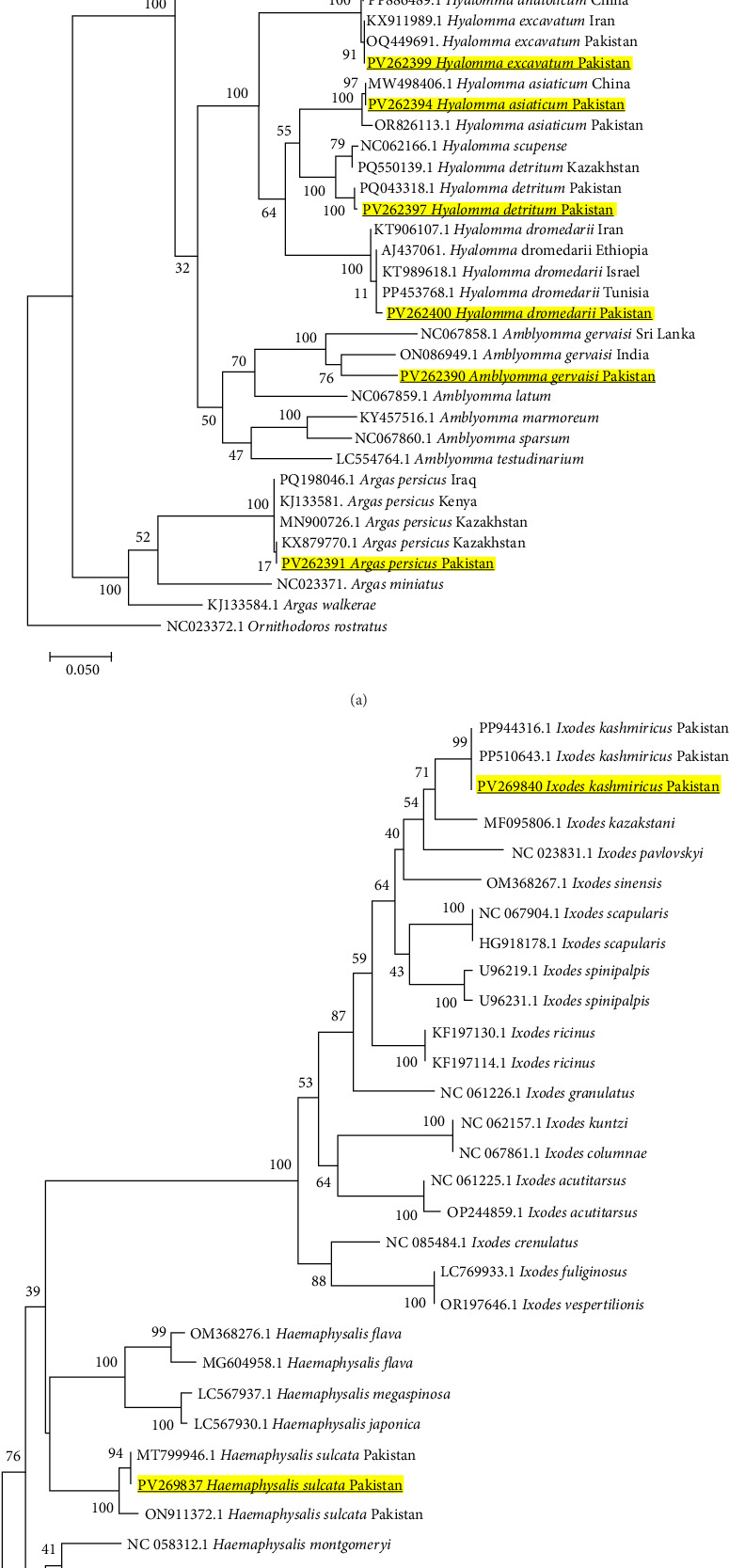
(A) Phylogeny of 13 tick species (Table S2) constructed using maximum likelihood estimations based on *COI* gene via applying Kimura 2-parameter model. Bold and yellow-highlighted sequences are characterized in the current study. Here, *Ornithodoros rostratus* (NC023372.1) was used as an outgroup. (B) Phylogeny of two tick species (Table S2) was constructed using the neighbor-joining method based on *16S rRNA* gene via applying the maximum composite likelihood model. Bold and yellow-highlighted sequences are characterized in the current study. Here, *Haemaphysalis humerosa* (JX573138.1) was used as an outgroup.

We optimized genetic markers (targeting *COI* and *16S rRNA* genes) for determining the phylogenetic relationship among all morphologically identified tick species. Based on these genetic markers, all the details of our obtained sequences regarding BLAST results, identities (%) with their corresponding isolates, reference accession numbers, and reported countries are shown in Table S2. BLAST identities based on *COI* gene of our obtained 13 sequences (PV262403-*Rh. sanguineus*, PV263164-*Rh. turanicus*, PV262401-*Rh. haemaphysaloides*, PV263162-*Rh. microplus*, PV263163-*Hae. bispinosa*, PV262392-*Hae. montgomeryi*, PV262393-*Hy. anatolicum*, PV262399-*Hy. excavatum*, PV262394-*Hy. asiaticum*, PV262397-*Hy. detritum*, PV262400-*Hy. dromedarii*, PV262390-*Am. gervaisi*, and PV262391-*Ar. persicus*) of different tick species ranged from 88.19% to 100% similarity index which made their separate cldes in phylogenetic tree with their respective corresponding homologous tick isolates as presented in Figure 2A. Additionally, the obtained sequences of *16S rRNA* gene (PV269840-*Ix. kashmiricus*, and PV269837-*Hae. sulcata*) showed 97.99%–100% similarities and clustered in phylogenetic tree with their corresponding tick species reported from Pakistan previously, as shown in Figure 2B.

### 3.5. Sequence Similarities and Phylogenetic Analysis of Tick-Borne Associated Microorganisms

We obtained a total of 18 consensus sequences for the detected TBMs based on various targeted genes. Overall, the obtained BLAST identity of all the obtained sequences based on consensus sequences of all genes was 98.17%–100%, while specific BLAST identities (%) based on each respective gene with corresponding isolates, along with reference accession numbers and reported countries are presented in (Table S3). Moreover, *R. slovaca* and *R. sibirica* were identified via four genes (*gltA*, *sca4*, *ompA*, and *ompB*) due to their novelty and first-time report in different tick species from Pakistan.

#### 3.5.1. *Anaplasma phagocytophilum* and *Coxiella burnetii*

**Figure 3:**
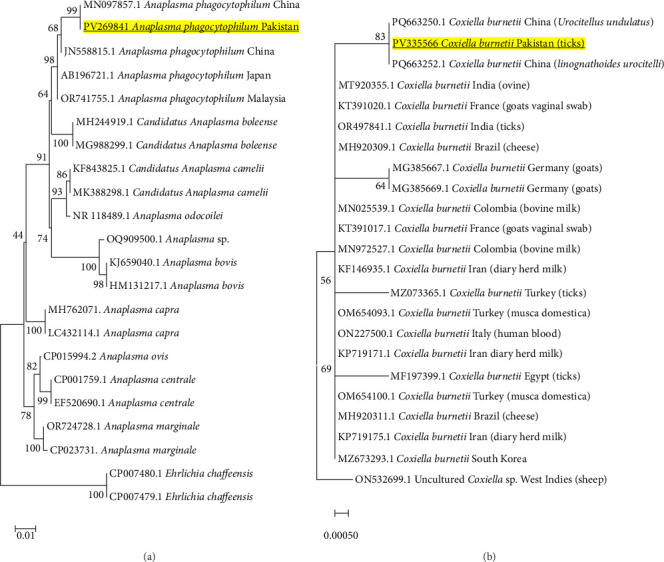
(A) Phylogeny of *Anaplasma phagocytophilum* (Table S3) constructed using neighbor-joining method based on *16S rRNA* gene via applying p-distance model. Bold and yellow-highlighted sequence (PV269841) is characterized in the current study. *Ehrlichia chaffeensis* (CP007480.1 and CP007479.1) was used as an outgroup. (B) Phylogeny of *Coxiella burnetii* (Table S3) constructed using neighbor-joining method based on *ISIIII* gene via applying Kimura 2-parameter method. Bold and yellow-highlighted sequence (PV335566) is characterized in the current study. Uncultured *Coxiella* sp. (ON532699.1) was used as an outgroup.

A phylogenetic tree was constructed based on the *16S rRNA* gene; the nucleotide sequences for *A. phagocytophilum* (PV269841) were retrieved, which shows that it grouped together with a strong bootstrap support with its corresponding isolates, as shown in Figure 3A. This high degree of genetic identity highlights the possible transboundary movement or a close epidemiological link of this pathogen. Similarly, the obtained sequence based on the *IS1111* gene for *C. burnetii* (PV335566) established a well-supported clade with other counterpart strains from China (PQ663250.1) and other geographical areas (Figure 3B). This suggests that common strains of *C. burnetii* genotypes are prevalent in the area, which is also shared with other regions of Asia and the world.

#### 3.5.2. *Hepatozoon* spp., *Babesia microti*, and *Theileria* spp.

**Figure 4:**
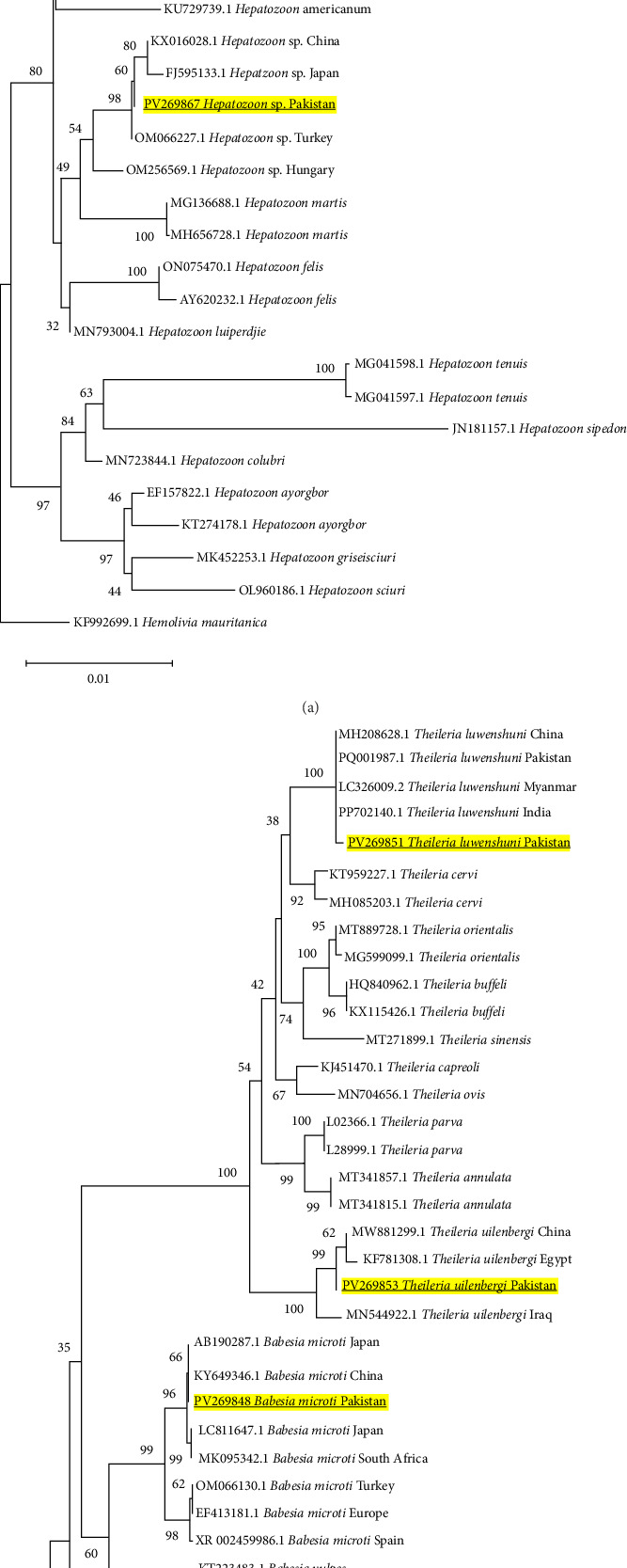
(A) Phylogeny of *Hepatozoon* spp. (Table S3) constructed using the neighbor-joining method based on the *18S rRNA* gene via applying the maximum composite likelihood model. Bold and yellow-highlighted sequences (PV269866 and PV269867) are characterized in the current study. *Hemolivia mauritanica* (KF992699.1) was used as an outgroup. (B) Phylogeny of *Theileria* spp. and *Babesia microti* (Table S3) was constructed using neighbor-joining method based on *18S rRNA* gene via applying the p-distance model. Bold and yellow-highlighted sequences (PV269848, PV269851, and PV269853) are characterized in the current study. *Babesia rodhaini* (MK256975.1 and DQ641423.1) was used as an outgroup.

Phylogenetic trees for *H. canis* (PV269866) and *Hepatozoon* sp. (PV269867) were constructed using *18S rRNA* gene sequences, revealing their clustering with similar isolates from China, Brazil, Malaysia, and other regions, contributing to the extensive distribution and genetic conservation of these parasites (Figure 4A). The *B. microti* sequence (PV269848), derived from the *18S rRNA* gene, clustered with comparable specimens from other regions, including China, Japan, and South Africa (MH206238.1), as illustrated in Figure 4B. The finding is noteworthy as it underscores the identification of a widely widespread *B. microti* genotype for the first time in ticks from Pakistan. Additionally, the sequences of *Th. luwenshuni* (PV269851) and *Th. uilenbergi* (PV269853) derived from *18S rRNA* gene were categorized within their respective clades, demonstrating close affiliations with strains documented from China, Myanmar, and India (Figure 4B). This corresponds with the established regional epidemiology of these ovine piroplasms.

#### 3.5.3. *Rickettsia* spp. (*R. slovaca*, *R. sibirica*, *R. raoultii*, *R. conorii* subsp. *raoultii*)

**Figure 5:**
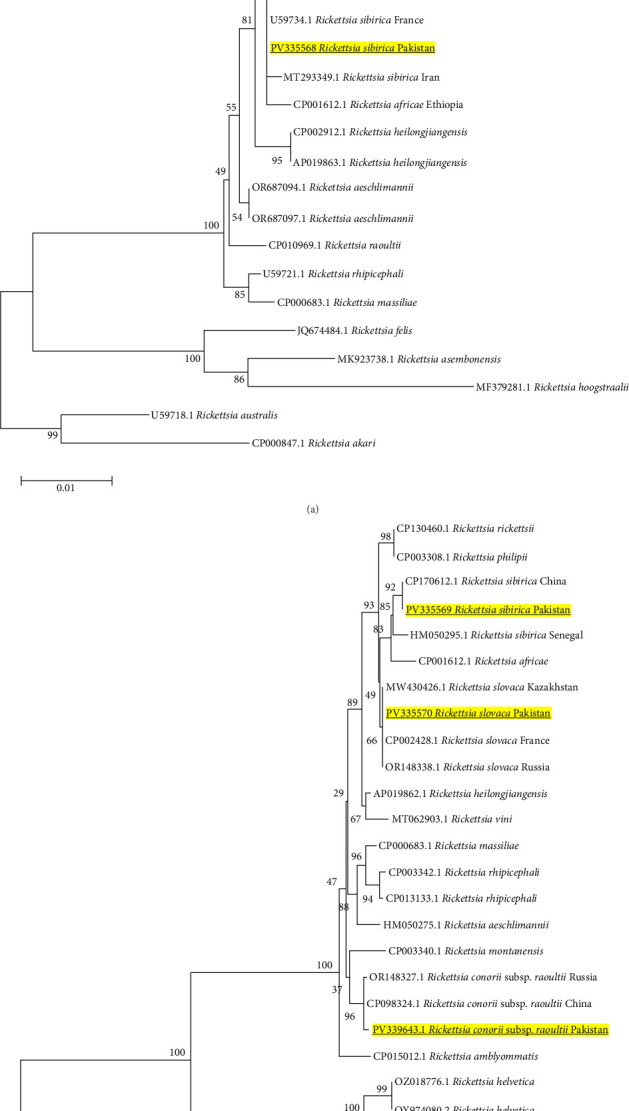
(A) Phylogeny of *Rickettsia* (*R*) spp. (Table S3) constructed using neighbor-joining method based on *gltA* gene via applying LogDet (Tamura-Kumar) model. Bold and yellow-highlighted sequences (PV335567 and PV335568) are characterized in the current study. *Rickettsia akari* (CP000847.1) and *Rickettsia australis* (U59718.1) were used as an outgroup. (B) Phylogeny of *Rickettsia* spp. (Table S3) constructed using neighbor-joining method based on *sca4* gene via applying LogDet (Tamura-Kumar) model. Bold and yellow-highlighted sequences (PV335569, PV335570, and PV339643) are characterized in the current study. *Rickettsia typhi* (CP003397.1) and *R. canadensis* (CP003304.1) were used as an outgroup.

**Figure 6:**
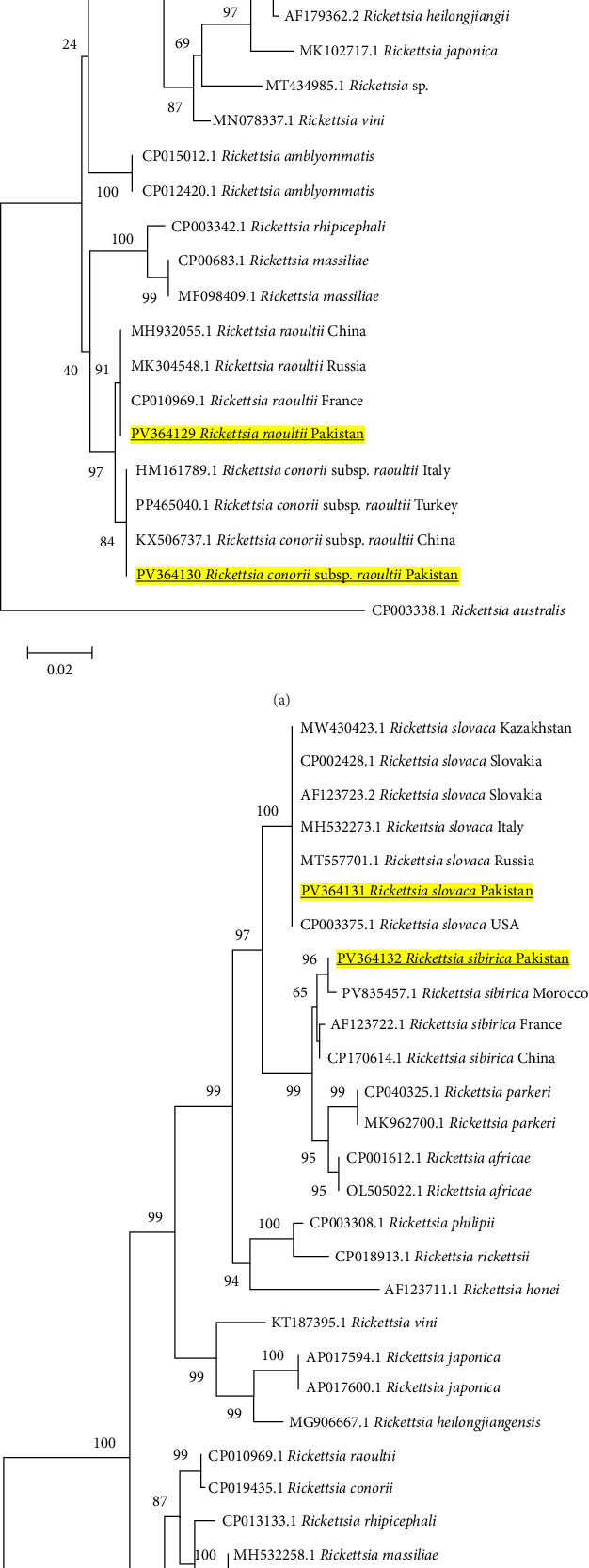
(A) Phylogeny of *Rickettsia* (*R*) spp. (Table S3) constructed using maximum likelihood method based on the *ompA* gene via applying Kimura 2-parameter model. Bold and yellow-highlighted sequences (PV364127, PV364128, PV364129, and PV364130) are characterized in the current study. *Rickettsia australis* (CP003338.1) was used as an outgroup. (B) Phylogeny of *Rickettsia* spp. (Table S3) constructed using neighbor-joining method based on *ompB* gene via applying maximum composite likelihood method. Bold and yellow-highlighted sequences (PV364131 and PV364132) are characterized in the current study. *Rickettsia felis* (AF182279.2) was used as an outgroup.

The phylogenetic investigation of *Rickettsia* species using various genes, including *gltA*, *sca4*, *ompA*, and *ompB*, revealed consistent and significant understanding concerning their genetic relationships. The novel finding of *R. slovaca* indicates that sequences generated from all four genes (*gltA*-PV335567, *sca4*-PV335570, *ompA*-PV364127, and *ompB*-PV364131) consistently grouped together alongside their respective strains previously reported from other countries, including France, Slovakia, Italy, and the USA (CP002428.1, MW779483.1) (Figures 5A,B and 6A,B) with strong bootstrap values. The strong genetic relationship indicates the presence of *R. slovaca* genotypes in Pakistan that resemble, with those identified in European areas, having zoonotic importance.

Moreover, *R. sibirica* sequences of the four genes (*gltA*-PV335568, *sca4*-PV335569, *ompA*-PV364128, and *ompB*-PV364132) consistently grouped with similar isolates from China, Russia, and Kazakhstan (CP003308.1, MT293349.1) (Figures 5A,B and 6A,B). This is significant since Pakistan is geographically close to these regions, and there is a likelihood that there might have been transboundary transmission of this pathogen either through migratory birds or trade in livestock. Furthermore, sequences of the *R. raoultii* (PV364129) and *R. conorii* subsp. *raoultii* (PV364130, PV339643) also clustered with species of their respective countries, including France, Russia, and Turkey (Figures 5B and 6A), suggesting they have a wide distribution and a conserved genome.

## 4. Discussion

The fascinating interplay of biological and environmental variables in Pakistan influences the distribution of resilient parasites (ticks) and associated microorganisms [40, 41, 43, 67]. To investigate ticks and TBPs in two provinces of Pakistan (Khyber Pakhtunkhwa and Punjab), a total of 15 tick species, including *Rh. microplus*, *Rh. haemaphysaloides*, *Rh. turanicus*, *Rh. sanguineus*, *Hy. anatolicum*, *Hy. asiaticum*, *Hy. dromedarii*, *Hy. detritum*, *Hy. excavatum*, *Hae. sulcata*, *Hae. bispinosa*, *Hae. montgomeryi*, *Ix. kashmiricus*, *Ar. persicus*, and *Am. gervaisi* were studied. Additionally, the identified associated TBMs include *R. sibirica*, *R. slovaca*, *R. raoultii*, *R. conorii* subsp. *raoultii*, *A. phagocytophilum*, *C. burnetii*, *H. canis*, *Hepatozoon* sp., *Th. luwenshuni*, *Th. uilenbergi*, and *B. microti*. This study documented the first-ever genetic evidence of two zoonotic *Rickettsia* spp., including *R. sibirica* and *R. slovaca*, and the zoonotic protozoan (*B. microti*) in various tick species from Pakistan.

Our findings regarding tick species composition, with a high prevalence of *Rh. microplus* (22.41%) aligns with previous reports from Pakistan [4, 40, 43, 68] but also resonates with data from neighboring countries such as India [69] and southern China [70], where it poses a significant threat to the livestock economy. The high incidence of this tick in Punjab province of Pakistan is comparable to that in the Indian state of Punjab, indicating that the two regions have similar climates and intensive livestock farming activities, which provide optimal conditions for tick's growth across the border [71]. The different environmental conditions in Pakistan and a considerable animal population, which serves as a mobile reservoir for these vectors, enable their widespread existence. Tick control in South and Central Asia is a regional challenge [72, 73], as evidenced by the dominance of the *Rhipicephalus* genus, which is also a major vector in Iran [74]. The epidemiological significance of *Rh. microplus* ticks in Pakistan are shown by our findings that 11.63% of them were infected with at least one pathogen. Due to its broad host range and primary parasitisation of cattle, this tick serves as an essential route for the spread of pathogens from livestock to possibly humans, which is evident from our finding of *R. slovaca* and *A. phagocytophilum* in *Rh. microplus*. Although *Dermacentor* ticks are considered as a common vector for *R. slovaca* in Europe [75], the finding about *Rhipicephalus* genera in the current study suggests an expanded transmission cycle in Pakistan, requiring further comprehensive investigation. Additionally, the finding of *R. sibirica* and *R. raoultii* in monitor lizard-collected tick species (*Am. gervaisi)* draw attention to a little-studied reptilian transmission cycle. Although the prevalence of *Am. gervaisi* was low in Punjab, which might be due to their habitat loss, its function as a reservoir for SFG rickettsiae cannot be overlooked, particularly in areas where human–wildlife interactions are increasingly common. Studies from other regions of Asia, where reptiles and their ectoparasites are becoming more widely acknowledged as elements of zoonotic disease cycles, are consistent with our findings [76].

The high prevalence of *C. burnetii* in the studied region could be due to its capacity to infect numerous tick species, including *Ar. persicus*, *Rh. microplus*, *Rh. haemaphysaloides*, *Hae. montgomeryi*, *Hy. anatolicum*, and *Hy. asiaticum*, which is in line with the previous findings [5, 77]. The finding of *C. burnetii* in multiple tick species emphasizes the need for additional research for the management of both hard and soft ticks in the region to mitigate zoonotic transmission, since several studies emphasized its huge global impact on human health [78–80].

Additionally, *Th luwenshuni* and *Th. uilenbergi* were identified with a prevalence rate of 0.28% and 0.39%, respectively. These protozoan pathogens infect sheep and goats and can be transmitted by the *Haemaphysalis* ticks, causing ovine theileriosis, especially in China and other regions of Asia [42, 81, 82], resulting in major economic losses [83]. In addition, *H. canis*, the causative agent of hepatozoonosis, infesting particularly canines, was detected at 0.55% prevalence in *Rh. sanguineus*, *Rh. turanicus*, and *Hy. excavatum* tick species, collected from dogs, which agrees with previous reports [29, 43, 84, 85], although its zoonotic nature is still undetermined [29]. Furthermore, *Babesia microti* is an emerging health threat causing human babesiosis, a malaria-like disease [86, 87]. This is the first molecular report confirming the detection of *B. microti* DNA in *Rh. haemaphysaloides*, *Hae. bispinosa*, and *Hy. asiaticum* ticks from Pakistan. This finding is of great epidemiological significance as previous studies in China, a neighboring country, have proven that *Rh. haemaphysaloides* and *Hy. asiaticum* can transmit this zoonotic pathogen [88]. In our investigation, the detection of its DNA with an overall prevalence of 0.33% in *Rh. haemaphysaloides*, *Hae. bispinosa*, and *Hy. asiaticum* ticks, highlighting the critical need for urgent surveillance and public awareness to reduce the impact of these novel pathogens. The widespread occurrence of tick infestations in domestic animals, including cattle, goats, and sheep, fosters an essential avenue for the transmission of zoonotic diseases. In the present study areas, conventional farming practices, including nomadic and transhumant herding, are widely practiced. The seasonal migration of animals across long distances fundamentally promotes the geographic propagation of infected ticks and pathogens into previously unexposed regions. Finding zoonotic pathogens like *C. burnetii* (Q-fever), *A. phagocytophilum*, *R. slovaca*, and *R. sibirica* in ticks collected from human companion animals highlights their immediate risk towards public health.

A comparative table regarding the documentation of *R. slovaca*, *R. sibirica*, and *B. microti* has been presented (Table 4). This study reveals the first molecular evidence of *R. slovaca*, *R. sibirica*, and *B. microti* in Pakistani ticks, indicating potential zoonotic concerns. It is particularly intriguing that *R. slovaca* has been identified: previously thought to be an endemic pathogen to Europe and related to TIBOLA/DEBONEL [14, 114–118], its presence in *Ix. kashmiricus* and *Rhipicephalus* spp. in Pakistan contribute to its geographic and vector expansion. This trend is consistent with migratory birds, which transfer ticks over vast distances or during the movement of livestock, a situation supported by the fact that *R. slovaca* is found worldwide in a variety of mammalian hosts. Furthermore, the 0.78% prevalence of *R. sibirica* in five tick species is significant epidemiologically because Pakistan is located near China and Central Asian countries, where this pathogen is widely distributed and causes Siberian tick typhus. *Rickettsia sibirica* may enter Pakistan through cross-border trade and animal movement across the northern mountainous borders shared with China [43, 119, 120]. The presence of such pathogen in ticks belonging to *Hyalomma* and *Rhipicephalus*, which are active biting ticks and feed on a diverse range of hosts, suggests a serious possibility of transmission to animals and humans. In the current study, phylogenetic inferences provides some clues of the vector competence, for example: the identified *R. slovaca* (PV335567, PV335570, PV364127, PV364131) and *R. sibirica* (PV335568, PV335569, PV364128, PV364132) from *Amblyomma*, *Ixodes*, *Hyalomma*, and *Rhipicephalus* ticks were grouped in the same clade with their European counterpart strains from its confirmed competent *Dermacentor* tick vector as illustrated in Figures 5A,B and 6A,B. Although we detected this pathogen in different tick species, their strong genetic association indicates the potential vector competencies in tick species at a local level contributing to the vector expansion. Additionally, phylogenetically *B. microti* sequence (PV269848) clustered with similar strains from China and Japan, as shown in Figure 4B, where *Rhipicephalus* and other hard ticks are involved in its transmission. The close phylogenetic relationship supports the idea that Pakistani tick species identified in present research are not simply accidental carriers; rather, they could potentially be competent vectors that play a significant role in the local ecology of these pathogens.

## 5. Conclusion

This study provides the first molecular evidence of *R. slovaca*, *R. sibirica*, *B. microti* in ticks from Pakistan, showing the country's trend as being an emerging hotspot for zoonotic TBPs. We reported 15 tick species from six genera, highlighting the complex vector-host-pathogen dynamics influenced by Pakistan's ecological diversity and livestock. Public health officials and the veterinary department should establish active surveillance programs of these emerging pathogens both in livestock and human populations, especially in rural areas where there is significant interactions between humans and animals. Moreover, the agriculture and livestock departments ought to formulate and distribute specific tick-control measures tailored for farmers and nomadic herders, including raising awareness about potential risks associated with TBDs and providing education concerning safe practices for handling animals and the use of acaricides. In addition, Physicians and testing centers must be vigilant regarding these emerging pathogens.

## Figures and Tables

**Table 1:** Details of various primers, used in current study for the identification of tick species and associated tick-borne microorganisms.

S. no.	Target organisms/gene	Primer name and sequence	Cycling conditions	Amplicon size	Reference
1	Tick/*COI* gene	LCO1490: GGTCAACAAATCATAAAGATATTGG	94°C for 3 min; 35x (94°C for 30 s, 51.7°C for 45 s, 72°C for 1 min); 72°C for 10 min	~710 bp	[50]
		HC02198: TAAACTTCAGGGTGACCAAAAAATCA			

2	Tick/*16S rRNA*	16S + 1: CCG GTC TGA ACT CAG ATC AAGT	94°C for 3 min; 35x (94°C for 30 s, 55°C for 30 s, 72°C for 1 min); 72°C for 10 min	~450 bp	[51]
		16S − 1: GCT CAA TGA TTT TTT AAA TTG CTG			

3	*Rickettsia* spp./*ompA*	Rr190.70p: ATGGCGAATATTTCTCCAAAA	94°C for 5 min; 35x (94°C for 1 min, 46°C for 30 s, 72°C for 1 min); 72°C for 10 min	~632 bp	[52]
		Rr190.701n: GTTCCGTTAATGGCAGCATCT			

4	*Rickettsia* spp./*ompB*	120- M59: CCGCAGGGTTGGTAACTGC	94°C for 5 min; 35x (94°C for 1 min, 56°C for 30 s, 72°C for 1 min); 72°C for 10 min	~862 bp	[53]
		120-807: CCTTTTAGATTACCGCCTAA			
5	*Rickettsia* spp./*sca4*	D1F: ATGAGTAAAGACGGTAACCT		~870 bp	[54]
		D928R: AAGCTATTGCGTCATCTCCG			
6	*Rickettsia* spp./*gltA*	CS-239: GCTCTTCTCATCCTATGGCTATTAT		~830 bp	[55]
		CS-1069: CAGGGTCTTCGTGCATTTCTT			
7	*Coxiella burnetii IS1111* gene	Trans 1: TATGTATCCACCGTAGCCA		~687 bp	[56]
		Trans 2: CCCAACAACACCTCCTTAT			
8	Piroplasm/*18S rRNA*	PIRO-A: ATAACCGTGCTAATTGTAGG		~450 bp	[57]
		PIRO-B: TGTTATTTCTTGTCACTACC			
9	*Anaplasma phagocytophilum/16S rRNA*	Inner-f SSAP2f: GCTGAATGTGGGGATAATTTAT		~641 bp	[58]
		Inner-r SSAP2r: ATGGCTGCTTCCTTTCGGTTA			

10	*Hepatozoon* spp./*18S rRNA*	HepF: ATACATGAGCAAAATCTCAAC	94°C for 5 min; 35x (94°C for 1 min, 49.5°C for 30 s, 72°C for 1 min); 72°C for 10 min	~643 bp	[59]
		HepR: CTTATTATTCCATGCTGCAG			

**Table 2:** District-wise distribution of various hosts and their associated tick species, including different life stages of ticks.

Study locations		Hosts			Morpho-molecularly identified tick species				
Provinces	Districts	Animal type	Examined (*n*)	Infested (*n*)	Tick species	Female (*F*)	Nymphs (*N*)	Males (*M*)	Total number of tick species from each district
Khyber Pakhtunkhwa	Chitral	Cattle	31	19	*Hyalomma* (*Hy*) *anatolicum*	9	11	3	23(13.29%)
					*Rhipicephalus* (*Rh*) *haemaphysaloides*	6	9	2	17(7.51%)
					*Rh. microplus*	11	15	3	29(16.76%)
		Camels	26	14	*Hy. dromedarii*	8	13	1	22(12.72%)
		Goats	27	15	*Rh. microplus*	5	7	0	12(6.94%)
					*Hy. asiaticum*	6	7	1	14(8.09%)
		Dogs	24	12	*Rh. sanguineus*	9	11	1	21(12.14%)
					*Hy. excavatum*	6	10	1	17(9.83%)
		Lizards	13	7	*Amblyomma* (*Am*) *gervaisi*	7	9	2	18(10.40%)
	Total		121 (9.04%)	67 (8.95%)	—	67/173 (38.73%)	92/173 (53.18%)	14/173 (8.09%)	173 (9.60%)
	Upper Dir	Cattle	30	17	*Rh. microplus*	12	17	2	31 (18.3%)
					*Hy. anatolicum*	9	14	2	25 (14.79%)
		Goats	28	16	*Hy. asiaticum*	6	11	1	18 (10.65%)
					*Ixodes* (*Ix*) *kashmiricus*	5	10	1	16 (9.7%)
		Sheep	27	14	*Haemaphysalis* (*Hae*) *sulcata*	5	11	1	17 (10.10%)
					*Hae. montgomeryi*	7	8	1	16 (9.7%)
		Dogs	26	15	*Hy. excavatum*	3	7	1	11 (6.51%)
					*Rh. sanguineus*	7	12	2	21 (12.3%)
		Lizards	12	6	*Am. gervaisi*	3	11	0	14 (8.28%)
	Total		123 (9.18%)	68 (9.08%)	—	57/169 (33.73%)	101/169 (59.76%)	11/169 (6.51%)	169 (9.37%)
	Malakand	Cattle	23	16	*Rh. microplus*	9	16	3	28 (10.84%)
		Camels	24	11	*Hy. anatolicum*	6	15	2	23 (13.86%)
					*Hy. dromedarii*	4	10	3	17 (10.24%)
		Goats	22	12	*Rh. microplus*	8	6	1	15 (9.03%)
					*Rh. haemaphysaloides*	5	13	1	19 (11.44%)
		Sheep	21	14	*Hae. montgomeryi*	6	5	1	12 (7.23%)
					*Ix. kashmiricus*	6	11	1	18 (10.84%)
		Dogs	21	12	*Rh. sanguineus*	8	12	1	21 (12.65%)
		Lizards	14	5	*Am. gervaisi*	7	5	1	13 (7.83%)
	Total		125 (9.33%)	70 (9.35%)	—	59/166 (35.54%)	93/166 (56.02%)	14/166 (8.43%)	166 (9.21%)
	Peshawar	Cattle	28	16	*Rh. haemaphysaloides*	4	14	1	19 (10.56%)
					*Rh. microplus*	8	19	2	29 (16.11%)
		Camels	19	11	*Hy. dromedarii*	5	16	1	22 (12.22%)
		Goats	22	13	*Hy. asiaticum*	6	11	1	18 (10.00%)
					*Rh. microplus*	6	7	1	14 (7.78%)
		Sheep	18	9	*Hae. montgomeryi*	4	11	1	16 (8.89%)
		Dogs	23	13	*Rh. sanguineus*	7	12	2	21 (11.67%)
					*Rh. turanicus*	6	13	0	19 (10.56%)
		Chicken	21	12	*Argas* (*Ar*) *persicus*	7	13	2	22 (12.22%)
	Total		131 (9.78%)	74 (9.88%)	—	53/180 (29.4%)	116/180 (64.4%)	11/180 (6.11%)	180 (9.98%)
	Lakki Marwat	Cattle	28	17	*Rh. microplus*	16	21	4	41 (23.98%)
		Camels	26	16	*Hy. dromedarii*	8	12	1	21 (12.28%)
					*Hy. asiaticum*	7	11	1	19 (11.11%)
					*Hy. anatolicum*	9	13	2	24 (14.03%)
		Goats	27	14	*Hae. bispinosa*	7	17	2	26 (15.20%)
		Dogs	23	11	*Rh. turanicus*	9	11	2	22 (12.86%)
		Chicken	15	8	*Ar. persicus*	6	11	1	18 (10.53%)
	Total		119 (8.89%)	66 (8.81%)	—	62/171 (36.25%)	96/171 (56.14%)	13/171 (7.60%)	171 (9.48%)
	South Waziristan	Cattle	25	15	*Rh. haemaphysaloides*	5	8	1	14 (8.33%)
					*Rh. microplus*	7	19	2	28 (16.67%)
		Camels	22	12	*Hy. dromedarii*	6	12	1	19 (11.31%)
					*Hy. asiaticum*	8	7	2	17 (10.12%)
		Goats	24	13	*Ix. kashmiricus*	7	9	2	18 (10.71%)
		Sheep	21	11	*Hae. bispinosa*	5	6	1	12 (7.14%)
					*Rh. sanguineus*	8	8	0	16 (9.52%)
		Dogs	17	12	*Hy. detritum*	7	11	1	19 (11.31%)
					*Hy. excavatum*	4	6	2	12 (7.14%)
		Chicken	18	8	*Ar. persicus*	7	6	0	13 (7.7%)
	Total		127 (9.48%)	71 (9.48%)	—	64/168 (38.09%)	92/168 (54.76%)	12/168 (7.14%)	168 (9.32%)

Punjab	Bahawalpur	Cattle	29	18	*Rh. microplus*	8	16	2	26 (15.95%)
					*Rh. haemaphysaloides*	7	13	1	21 (12.88%)
					*Hy. anatolicum*	5	12	0	17 (10.34%)
		Camels	23	13	*Hy. asiaticum*	8	10	1	19 (11.66%)
					*Hy. dromedarii*	7	7	1	15 (9.20%)
		Goats	25	15	*Rh. microplus*	4	8	0	12 (7.36%)
					*Hy. detritum*	7	11	1	19 (11.66%)
		Sheep	21	12	*Hae. bispinosa*	4	13	2	19 (11.66%)
		Dogs	18	7	*Rh. sanguineus*	4	11	0	15 (9.20%)
	Total		116 (8.66%)	65 (8.67%)	—	54/163 (33.13%)	101/163 (61.96%)	8/163 (4.91%)	163 (9.04%)
	Muzaffargarh	Cattle	28	17	*Rh. haemaphysaloides*	7	11	1	19 (12.50%)
					*Rh. microplus*	9	18	0	27 (17.76%)
		Camels	23	12	*Hy. asiaticum*	7	6	1	14 (9.21%)
					*Hy. dromedarii*	7	14	1	22 (14.7%)
		Goats	25	15	*Ix. kashmiricus*	4	7	0	11 (7.24%)
					*Rh. turanicus*	7	13	1	21 (13.82%)
		Sheep	26	16	*Hae. bispinosa*	4	17	2	23 (15.13%)
		Chicken	15	5	*Ar. persicus*	4	9	0	13 8.55%)
	Total		117 (8.73%)	65 (8.67%)	—	49/150 (32.67%)	95/150 (63.33%)	6/150 (4.00%)	150 (8.32%)
	Lodhran	Cattle	29	19	*Rh. microplus*	11	14	3	28 (18.92%)
					*Rh. haemaphysaloides*	3	8	0	11 (7.43%)
					*Hy. anatolicum*	4	10	1	15 (10.14%)
		Goats	28	17	*Hy. detritum*	9	14	2	25 (16.89%)
		Sheep	25	14	*Hae. sulcata*	7	13	2	22 (14.86%)
		Dogs	24	13	*Rh. turanicus*	4	14	1	19 (12.84%)
					*Hy. excavatum*	3	12	1	16 (10.81%)
		Chicken	16	6	*Ar. persicus*	5	7	0	12 (8.11%)
	Total		122 (9.11%)	69 (9.21%)	—	46/148 (31.08%)	92/148 (62.16%)	10/148 (7.76%)	148 (8.21%)
	Rahim Yar Khan	Cattle	26	16	*Rh. haemaphysaloides*	4	13	1	18 (11.92%)
					*Rh. microplus*	9	22	0	31 (20.53%)
		Camels	25	15	*Hy. asiaticum*	3	7	1	11 (7.28%)
					*Hy. dromedarii*	7	14	0	21 (13.91%)
		Goats	28	17	*Hae. sulcata*	3	10	1	14 (9.27%)
					*Rh. microplus*	5	15	1	21 (13.91%)
		Dogs	22	10	*Rh. turanicus*	7	14	0	21 (13.91%)
		Chicken	17	8	*Ar. persicus*	6	8	0	14 (9.27%)
	Total		118 (8.81%)	66 (8.82%)	—	44/151 (29.14%)	103/151 (68.21%)	4/151 (2.65%)	151 (8.37%)
	Vehari	Cattle	27	16	*Rh. haemaphysaloides*	3	11	1	15 (9.15%)
					*Rh. microplus*	11	17	4	32 (19.51%)
		Camels	22	14	*Hy. dromedarii*	7	3	1	11 (6.71%)
					*Hy. anatolicum*	13	12	2	27 (16.46%)
		Goats	28	17	*Ix. kashmiricus*	8	14	1	23 (14.02%)
		Sheep	23	10	*Hae. sulcata*	10	14	2	26 (15.85%)
		Dogs	20	11	*Rh. turanicus*	6	8	0	14 (8.54%)
					*Hy. detritum*	4	11	1	16 (9.76%)

	Total		120 (8.96%)	68 (9.08%)	*Ar. persicus* = 92/1803(5.10%) *Rh. microplus* = 404/1803 (22.41%) *Rh. turanicus* = 116/103 (6.43%) *Rh. sanguineus* = 115/1803 (6.37%) *Rh. haemaphysaloides* = 153/1803 (8.48%) *Hae. bispinosa =* 80/1803 (4.44%) *Hae. sulcata* = 79/1803 (4.3%) *Hae. montgomeryi* = 44/1803 (2.44%) *Hy. dromedarii* = 170/1803 (9.43%) *Hy. detritum* = 79/1803 (4.38%) *Hy. excavatum* = 56/1803 (3.11%) *Hy. anatolicum* = 154/1803 (8.54%) *Ix. kashmiricus* = 86/1803 (4.77%) *Hy. asiaticum* = 130/1803 (7.21%) *Am. gervaisi* = 45/1803 (2.49%)	62/164 (37.80%)	90/164 (54.88%)	12/164 (7.32%)	164 (9.09%)
Overall			1339	749 (55.94%)		617/1803 (34.22%)	1071/1803 (59.40%)	115/1803 (6.38%)	1803
Detailed infested host animals and tick species were:		Cattle = 186/749 (24.83%) Goats = 164/749 (21.89%) Camels = 118/749 (15.75%) Dogs = 116/749 (15.49%) Sheep = 100/749 (13.35%) Chickens = 47/749 (6.27%) Lizards = 18/749 (2.40%)							
							—	—	—

**Table 3:** Prevalence and distribution rates of associated TBMs in individual tick species.

Tick species	Prevalence rate of each associated TBMs in each tick species											Total positivity rates of each tick species for their associated TBMs (%)	Genus-based prevalence rate for overall TBMs
	Rickettsiales bacteria (%)					*Coxiella burnetii* (%)	*Hepatozoon* (*%*)		Piroplasms (%)				
	*Rickettsia* (*R*) *sibirica*	*R. slovaca*	*R. raoultii*	*R. conorii* subsp. *raoultii*	*Anaplasma phagocytophilum*		*Hepatozoon* sp.	*Hepatozoon canis*	*Theileria* (*Th*) *luwenshuni*	*Th. uilenbergi*	*Babesia microti*		
*Argas persicus*	—	—	2.17% (2/92; 95% CI: 0.49%−7.39%)	—	—	3.26% (3/92; 95% CI: 0.89%−9.19%)	—	—	—	—	—	5.43% (5/92; 95% CI: 1.83%−11.96%)	5.43% (5/92; 95% CI: 1.83%−11.96%)

*Rhipicephalus* (*Rh*) *microplus*	—	2.97% (12/404; 95% CI: 1.63%−5.11%)	—	—	4.46% (18/404; 95% CI: 2.72%−7.01%)	2.23% (9/404; 95% CI: 1.07%−4.30%)	—	—	0.74% (3/404; 95% CI: 0.19%−2.16%)	1.24% (5/404; 95% CI: 0.44%−2.84%)	—	11.63% (47/404; 95% CI: 8.76%−15.19%)	10.28% (81/788; 95% CI: 8.24%−12.68%)
*Rh. turanicus*	2.59% (3/116; 95% CI: 0.74%−7.23%)	—	—	4.35% (5/116; 95% CI: 1.49%−9.82%)	—	—	—	2.59% (3/116; 95% CI: 0.74%−7.23%)	—	—	—	11.63% (47/404; 95% CI: 8.76%−15.19%)	
*Rh. sanguineus*	—	—	4.35% (5/115; 95% CI: 1.49%−9.82%)	—	—	—	—	2.61% (3/115; 95% CI: 0.75%−7.28%)	—	—	—	6.96% (8/115; 95% CI: 3.06%−13.19%)	
*Rh. haemaphysaloides*	1.96% (3/153; 95% CI: 0.56%−5.60%)	1.31% (2/153; 95% CI: 0.28%−4.66%)	—	—	—	3.27% (5/153; 95% CI: 1.25%−7.46%)	1.96% (3/153; 95% CI: 0.56%−5.60%)	—	—	—	1.31% (2/153; 95% CI: 0.28%−4.66%)	9.80% (15/153; 95% CI: 5.92%−15.34%)	

*Haemaphysalis* (*Hae*)*bispinosa*	—	—	2.50% (2/80; 95% CI: 0.57%−8.77%)	—	—	—	1.25% (1/80; 95% CI: 0.06%−6.84%)	—	2.50% (2/80; 95% CI: 0.57%−8.77%)	—	2.50% (2/80; 95% CI: 0.57%−8.77%)	8.75% (7/80; 95% CI: 3.86%−17.65%)	4.93% (10/203; 95% CI: 2.57%−9.20%)
*Hae. sulcata*	—	—	—	—	—	—	—	—	—	—	—	0.00% (0/79; 95% CI: 0.00%−4.57%)	
*Hae. montgomeryi*	—	—	—	2.27% (1/44; 95% CI: 0.11%−12.28%)	—	2/44 (4.55%)	—	—	—	—	—	6.82% (3/44; 95% CI: 1.87%−18.06%)	

*Hyalomma* (*Hy*) *dromedarii*	2.94% (5/170; 95% CI: 1.14%−6.85%)	—	—	—	3.52% (6/170; (CI: 1.48%−7.70%)	—	—	—	—	—	—	6.47% (11/170; 95% CI: 3.55%−11.41%)	9.85% (48/589; 95% CI: 7.42%−12.92%)
*Hy. detritum*	—	3.80% (3/79; 95% CI: 1.03%−10.43%)	—	—	—	—	2.53% (2/79; 95% CI: 0.58%−8.88%)	—	—	—	—	6.33% (5/79; 95% CI: 2.15%−13.88%)	
*Hy. excavatum*	—	—	—	—	—	—	—	7.14% (4/56; 95% CI: 2.21%−16.79%)	—	—	—	7.14% (4/56; 95% CI: 2.21%−16.79%)	
*Hy. anatolicum*	—	3.25% (5/154; 95% CI: 1.25%−7.42%)	—	—	—	5.84% (9/154; 95% CI: 3.01%−10.74%)	—	—	—	1.30% (2/154; 95% CI: 0.28%−4.63%)	—	10.39% (16/154; 95% CI: 6.55%−15.93%)	
*Hy. asiaticum*	1.54% (2/130; 95% CI: 0.33%−5.46%)	—	—	2.31% (3/130; 95% CI: 0.66%−6.72%)	1.54% (2/130; 95% CI: 0.33%−5.46%)	2.31% (3/130; 95% CI: 0.66%−6.72%)	—	—	—	—	1.54% (2/130; 95% CI: 0.33%−5.46%)	9.23% (12/130; 95% CI: 5.17%−15.86%)	

*Ixodes kashmiricus*	—	4.65% (4/86; 95% CI: 1.53%−11.23%)	—	—	—	—	2.33% (2/86; 95% CI: 0.53%−8.15%)	—	—	—	—	6.98% (6/86; 95% CI: 2.87%−14.86%)	6.98% (6/86; 95% CI: 2.87%−14.86%)

*Amblyomma gervaisi*	2.22% (1/45; 95% CI: 0.11%−12.00%)	—	4.44% (2/45; 95% CI: 1.02%−14.59%)	—	—	—	—	—	—	—	—	6.67% (3/45; 95% CI: 1.83%−17.75%)	6.67% (3/45; 95% CI: 1.83%−17.75%)

Overall prevalence rate of each TBMs in collected ticks	4.77% (86/1803; 95% CI: 3.82%−5.91%)	1.44% (26/1803; 95% CI: 0.94%−2.09%)	0.61% (11/1803; 95% CI: 0.32%−1.09%)	0.50% (9/1803; 95% CI: 0.23%−0.96%)	1.44% (26/1803; 95% CI: 0.94%−2.09%)	1.72% (31/1803; 95% CI: 1.18%−2.43%)	0.44% (8/1803; 95% CI: 0.19%−0.87%)	0.55% (10/1803; 95% CI: 0.26%−1.04%)	0.28% (5/1803; 95% CI: 0.09%−0.66%)	0.39% (7/1803; 95% CI: 0.16%−0.79%)	0.33% (6/1803; 95% CI: 0.12%−0.72%)	8.48% (153/1803; 95% CI: 7.27%−9.82%)	
Total prevalence (%)	4.77% (86/1803; 95% CI: 3.82%−5.91%)						0.99% (18/1803; 95% CI: 0.59%−1.63%)		0.99% (18/1803; 95% CI: 0.59%−1.63%)				

**Table 4:** Details regarding the documentation of *Rickettsia* (*R*) *slovaca*, *Rickettsia sibirica*, and *Babesia microti* and their associated host vectors/sources in various countries.

Tick-borne pathogens	Reported country	Reported hosts/source	Study references
*Rickettsia slovaca*	Portugal	*Dermacentor* (*De*) *marginatus*	[89]
	Turkey	*De. marginatus*	[90]
	Turkey	*De. marginatus*	[91]
	Turkey	*De. marginatus*	[92]
	Russia	*De. reticulatus*	[93]
	Spain	*De. reticulatus*	[94]
		*Hyalomma* (*Hy*) *marginatum*	
		*Ixodes* (*Ix*) *ricinus*	
	Kazakhstan	*De. marginatus*	[95]
	Ukraine	*De. marginatus*	[96]
	Iran	*De. marginatus*	[97]
	Iran	*Rh. sanguineus*	[98]
	Kyrgyzstan	*Dermacentor* sp.	[99]
	China	*Melophagus ovinus*	[100]

*Rickettsia sibirica*	Morocco	*Hy. aegyptium*	[101]
	China	*Hy. asiaticum*	[102]
	China	*De. nuttalli*	[7]
	China	*De. sinicus*	[6]
		*Haemaphysalis* (*Hae*) *yeni*	
	Kenya	*Rhipicephalus* (*Rh*) *evertsi*	[103]
	Kenya	*Rh. appendiculatus*	[104]
	Russia	*De. reticulatus*	[105]
	Iran	*Rh. bursa*	[97]

*Babesia microti*	Republic of Korea	*Amblyomma* (*Am*) *testudinarium*	[106]
		*Hae. longicornis*	
	Italy	*Ix. ricinus*	[107]
	Ukraine	*Ix. ricinus*	[108]
	Mongolia	*Ix. persulcatus*	[109]
	Poland	*De. reticulatus*	[110]
	Turkey	*Hy. marginatum*	[111]
	Hungary	*Ix. ricinus*	[112]
	Germany	*Ix. ricinus*	[113]

## Data Availability

All the data can be freely available in online databases. Accession numbers of *COI* gene (*Amb. gervaisi*-PV262390, *Ar. persicus*-PV262391, *Hae. bispinosa*-PV263163, *Hae. montgomeryi*-PV262392, *Hy. anatolicum*-PV262393, *Hy. asiaticum*-PV262394, *Hy. detritum*-PV262397, *Hy. dromedarii*-PV262400, *Hy. excavatum*-PV262399, *Rh. microplus*-PV263162, *Rh. haemaphysaloides*-PV262401, *Rh. sanguineus*-PV262403, *Rh. turanicus*-PV263164), 16S rRNA gene (*Hae. sulcata*- PV269837,*Ix. kashmiricus*- PV269840, *A. phagocytophilum*-PV269841), *ISIIII* gene (*C. burnetii*-PV335566), *18S rRNA* gene (*H. canis*-PV269866, *Hepatozoon* sp.-PV269867, *B. microti*-PV269848, *Th. luwenshuni*-PV269851, *Th. uilenbergi*-PV269853), *gltA* gene (*R. slovaca*-PV335567, *R. sibirica*-PV335568), *sca4* gene (*R. slovaca*-PV335570, *R. sibirica*-PV335569, *R. conorii* subsp. *raoultii*-PV339643), *ompA* gene (*R. slovaca*-PV364127, *R. sibirica*-PV364128, *R. raoultii*-PV364129, *R. conorii* subsp. *raoultii*-PV364130), and *ompB* gene (*R. slovaca*-PV364131, *R. sibirica*-PV364132) can be found freely in NCBI (GenBank; http://www.ncbi.nlm.nih.gov/genbank/).

## Supporting Information

Supporting Information Table S1. Ticks load per animal and host-wise distribution with overall prevalence rates of each tick species. Table S2. Obtained accession numbers based on the *COI* gene with their BLAST identity, corresponding isolates' accession numbers, and reported country. Table S3. Obtained accession numbers based on *16S rRNA*, *ISIIII*, *18S rRNA*, *gltA*, *sca4*, *ompA*, and *ompB* genes with their BLAST identity, corresponding isolates' accession numbers, and reported country.
