# Supplementary material for: Molecular Profiling of Ticks and Associated Pathogens: First Report of Rickettsia sibirica, Rickettsia slovaca, and Babesia microti in Ticks From Pakistan
Source: Transbound Emerg Dis. 2025 Oct 6;2025:3157047. doi: 10.1155/tbed/3157047 (PMC12517992; doi:10.1155/tbed/3157047)
Supplement: Supporting Information — Table S1. Ticks load per animal and host-wise distribution with overall prevalence rates of each tick species. Table S2. Obtained accession numbers based on the COI gene with their BLAST identity, corresponding isolates' accession numbers, and reported country. Table S3. Obtained accession numbers based on 16S rRNA, ISIIII, 18S rRNA, gltA, sca4, ompA, and ompB genes with their BLAST identity, corresponding isolates' accession numbers, and reported country. [file 3157047.f1.docx]

**Molecular Profiling of Ticks and Associated Pathogens: First Report of Rickettsia sibirica, Rickettsia slovaca, and Babesia microti in ticks from Pakistan**

Muhammad Kashif Obaid^1^**^⁎^**, Jin Luo^1^, Shuaiyang Zhao^1^, Zhancheng Tian^1^, Shakir Ullah^2^, Jehan Zeb^3^, Guangyuan Liu^1^, Jianxun Luo^1^, Hong Yin^1^, Muhammad Rashid^4^, Qiaoyun Ren ^1,5^**^⁎^**, Guiquan Guan^1^**^⁎^**.

**^1^** State Key Laboratory for Animal Disease Control and Prevention, Key Laboratory of Veterinary, Parasitology of Gansu Province, Lanzhou Veterinary Research Institute, Chinese Academy of Agricultural Sciences, Lanzhou, Gansu, China ([kashifobaidkanz@gmail.com](mailto:kashifobaidkanz@gmail.com), [luojin02@caas.cn](mailto:luojin02@caas.cn), [zhaoshuaiyang@caas.cn](mailto:zhaoshuaiyang@caas.cn), [tianzhancheng@caas.cn](mailto:tianzhancheng@caas.cn), [liuguangyuan2002@sina.com](mailto:liuguangyuan2002@sina.com), [luojianxun@caas.cn](mailto:luojianxun@caas.cn), [yinhong@caas.cn](mailto:yinhong@caas.cn), [ren_qiaoyun@126.com](mailto:ren_qiaoyun@126.com), [guanguiquan@caas.cn](mailto:guanguiquan@caas.cn))

**^2^** Department of Zoology, Abdul Wali Khan University Mardan, Pakistan ([ullahshakir868@gmail.com](mailto:ullahshakir868@gmail.com))

**^3^** School of Public Health, The University of Hong Kong, Hong Kong SAR, Hong Kong, China ([zebjehan2012@gmail.com](mailto:zebjehan2012@gmail.com))

**^4^** Department of Parasitology, Faculty of Veterinary and Animal Sciences, The Islamia University of Bahawalpur, Punjab 63100, Pakistan ([rashid.laghari@iub.edu.pk](mailto:rashid.laghari@iub.edu.pk))

**^5^** Hebei Key Laboratory of Animal Physiology, Biochemistry and Molecular Biology, Hebei Collaborative Innovation Center for Eco-Environment, Ministry of Education Key Laboratory of Molecular and Cellular Biology, College of Life Sciences, Hebei Normal University, Shijiazhuang 050024, China ([ren_qiaoyun@126.com](mailto:ren_qiaoyun@126.com))

**Corresponding Authors**: Guiquan Guan ([guanguiquan@caas.cn](mailto:guanguiquan@caas.cn))

Qiaoyun Ren ([ren_qiaoyun@126.com](mailto:ren_qiaoyun@126.com))

Muhammad Kashif Obaid ([kashifobaidkanz@gmail.com](mailto:kashifobaidkanz@gmail.com))

**S-Table 1.** Ticks load per animal and host-wise distribution with overall prevalence rates of each tick species.

| **Tick Species** | **Infested hosts** | | | | | | | **Overall prevalence rate of each tick species (%)** |
| --- | --- | --- | --- | --- | --- | --- | --- | --- |
|  | **Cattle** | **Camels** | **Goats** | **Sheep** | **Dogs** | **Chicken** | **Lizards** |  |
| *Argas persicus* | - | - | - | - | - | 92/92 (100.00%) | - | **92 (5.10%)** |
| *Rhipicephalus (Rh) microplus* | 330/404 (81.68%) | - | 74/404 (18.32%) | - | - | - | - | **404 (22.41%)** |
| *Rh. turanicus* | - | - | 21/116 (18.10%) | - | 95/116 (81.89%) | - | - | **116 (6.43%)** |
| *Rh. sanguineus* | - | - | - | 16/115 (13.91%) | 99/115 (86.09%) | - | - | **115 (6.38%)** |
| *Rh. haemaphysaloides* | 134/153 (87.58%) | - | 19/153 (12.42%) | - | - | - | - | **153 (8.8%)** |
| *Haemaphysalis* *(Hae) bispinosa* | - | - | 26/80 (32.50%) | 54/80 (67.50%) | - | - | - | **80 (4.44%)** |
| *Hae. sulcata* | - | - | 14/79 (17.72%) | 65/79 (82.28%) | - | - | - | **79 (4.38%)** |
| *Hae. montgomeryi* | - | - | - | 44/44 (100.00%) | - | - | - | **44 (2.44%)** |
| *Hyalomma (Hy) dromedarii* | - | 170/170 (100.00%) | - | - | - | - | - | **170 (9.43%)** |
| *Hy. detritum* | - | - | - | 44/79 (55.69%) | 35/79 (44.30%) | - | - | **79 (4.38%)** |
| *Hy. excavatum* | - | - | - | - | 56/56 (100.00%) | - | - | **56 (3.11%)** |
| *Hy. anatolicum* | 80/154 (51.95%) | 74/154 (48.05%) | - | - | - | - | - | **154 (8.54%)** |
| *Hy. asiaticum* | - | 80/130 (61.54%) | 50/130 (38.6%) | - | - | - | - | **130 (7.21%)** |
| *Ixodes kashmiricus* | - | - | 68/86 (79.07%) | 18/86 (20.93%) | - | - | - | **86 (4.77%)** |
| *Amblyomma gervaisi* | - | - | - | - | - | - | 45/45 (100.00%) | **45 (2.49%)** |
| **Host-wise overall distribution of ticks** | **544/1803 (30.17%)** | **324/1803 (17.97%)** | **272/1803 (15.09%)** | **241/1803 (13.37%)** | **285/1803 (15.81%)** | **92/1803 (5.10%)** | **45/1803 (2.49%)** | **1803** |
| **Ticks load per animal (host based)** | **544/186 (2.92)** | **324/118 (2.75)** | **272/164 (1.66)** | **241/100 (2.41)** | **285/116 (2.46)** | **92/47 (1.96)** | **45/18 (2.50)** | **1803/749 (2.41)** |

**S-Table 2.** Obtained accession numbers based on COI gene with their BLAST identity, corresponding isolates accession numbers, and reported country.

| **S. No.** | **Targeted gene** | **Own obtained accession no.** | **Corresponding isolate(s)** | | | | **Figure no.?** |
| --- | --- | --- | --- | --- | --- | --- | --- |
|  |  |  | **Tick specie(s)** | **BLAST Identity** | **Accession no.** | **reported from** |  |
| 1 | *COI* gene | PV262390 | *Amblyomma. gervaisi* | 88.19─91.65% | NC_067858.1 | Sri Lanka | Figure 2 A |
|  |  |  |  |  | ON086949.1 | India |  |
| 2 |  | PV262391 | *Argas (Ar) persicus* | 99.85─100% | PQ198046.1 | Iraq |  |
|  |  |  |  |  | KJ133581.2 | Kenya |  |
|  |  |  |  |  | MN900726.1 | Kazakhstan |  |
|  |  |  |  |  | KX879770.1 |  |  |
| 3 |  | PV263163 | *Haemaphysalis (Hae) bispinosa* | 99.85─100% | NC_071765.1 | China |  |
|  |  |  |  |  | OR742954.1 | Malaysia |  |
|  |  |  |  |  | PQ687485.1 | India |  |
| 4 |  | PV262392 | *Hae. montgomeryi* | 90.83─97.42% | OM469326.1 | Pakistan |  |
|  |  |  |  |  | NC_058312.1 | China |  |
| 5 |  | PV262393 | *Hyalomma (Hy). anatolicum* | 99.69─100% | PP886489.1 | China |  |
|  |  |  |  |  | MK462203.1 | Pakistan |  |
| 6 |  | PV262394 | *Hy. asiaticum* | 98.85─100% | MW498406.1 | China |  |
|  |  |  |  |  | OR826113.1 | Pakistan |  |
| 7 |  | PV262397 | *Hy. detritum* | 97.01─99.84% | PQ043318.1 | Pakistan |  |
|  |  |  |  |  | PQ550139.1 | Kazakhstan |  |
| 8 |  | PV262400 | *Hy. dromedarii* | 99.85─100% | PP453768.1 | Tunisia |  |
|  |  |  |  |  | KT989618.1 | Israel |  |
|  |  |  |  |  | AJ437061.1 | Ethiopia |  |
|  |  |  |  |  | KT906107.1 | Iran |  |
| 9 |  | PV262399 | *Hy. excavatum* | 100% | KX911989.1 | Iran |  |
|  |  |  |  |  | OQ449691.1 | Pakistan |  |
| 10 |  | PV263162 | *Rhipicephalus (Rh) microplus* | 99.42─100% | OR241102.1 | India |  |
|  |  |  |  |  | PQ385874.1 | Pakistan |  |
|  |  |  |  |  | MW751680.1 | China |  |
| 11 |  | PV262401 | *Rh. haemaphysaloides* | 98.65─100% | PQ433525.1 | India |  |
|  |  |  |  |  | MT800317.1 | Pakistan |  |
| 12 |  | PV262403 | *Rh. sanguineus* | 99.84─99.85% | PQ425331.1 | Cameroon |  |
|  |  |  |  |  | MK648406.1 |  |  |
| 13 |  | PV263164 | *Rh. turanicus* | 99.29─99.55% | MT800314.1 | Pakistan |  |
|  |  |  |  |  | OR826115.1 |  |  |
| 14 | *16S rRNA* | PV269837 | *Hae. sulcata* | 97.99─100% | ON911372.1 | Pakistan | Figure 2 B |
|  |  |  |  |  | MT799946.1 |  |  |
| 15 |  | PV269840 | *Ixodes kashmiricus* | 100% | PP510643.1 | Pakistan |  |
|  |  |  |  |  | PP944316.1 |  |  |

**S-Table 3.** Obtained accession numbers based on *16S rRNA*, *ISIIII*, *18S rRNA*, *gltA*, *sca4*, *ompA*, *ompB* genes with their BLAST identity, corresponding isolates accession numbers, and reported country.

| **S. No.** | **Targeted gene** | **Own obtained accession no.** | **Corresponding isolate(s)** | | | |  |
| --- | --- | --- | --- | --- | --- | --- | --- |
|  |  |  | **TBM (organism)** | **BLAST Identity** | **Accession no.** | **Reported from** | **Figure no?** |
| **1** | *16S rRNA* | PV269841 | *Anaplasma phagocytophilum* | 100% | MN097857.1 | China | Figure 3 A,B |
| **2** | *ISIIII* gene | PV335566 | *Coxiella burnetii* | 100% | PQ663252.1 | China |  |
|  |  |  |  |  | PQ663250.1 |  |  |
| **3** | *18S rRNA* | PV269866 | *Hepatozoon canis* | 98.45–100% | MT107097.1 | China | Figure 4 A,B |
|  |  |  |  |  | AY461375.2 | Brazil |  |
|  |  |  |  |  | KU729737.1 |  |  |
|  |  |  |  |  | KT267961.1 | Malaysia |  |
|  |  |  |  |  | LC169075.2 | Japan |  |
|  |  |  |  |  | MN393911.1 | Cuba |  |
|  |  |  |  |  | KX712123.1 | Austria |  |
|  |  |  |  |  | FJ497022.1 | Croatia |  |
|  |  |  |  |  | KJ572976.1 | Hungary |  |
|  |  |  |  |  | OP587282.1 | Poland |  |
|  |  |  |  |  | PV077333.1 | Italy |  |
|  |  |  |  |  | LC331053.1 | Zambia |  |
|  |  |  |  |  | MH615006.1 | Israel |  |
|  |  |  |  |  | KX712126.1 | Romania |  |
| **4** | *18S rRNA* | PV269867 | *Hepatozoon* sp. | 98.59–100% | KX016028.1 | China |  |
|  |  |  |  |  | FJ595133.1 | Japan |  |
|  |  |  |  |  | OM066227.1 | Turkey |  |
|  |  |  |  |  | OM256569.1 | Hungary |  |
| **5** | *18S rRNA* | PV269848 | *Babesia microti* | 99.77–100% | AB190287.1 | Japan |  |
|  |  |  |  |  | KY649346.1 | China |  |
|  |  |  |  |  | MK095342.1 | South Africa |  |
| **6** | *18S rRNA* | PV269851 | *Theileria (Th) luwenshuni* | 99.77% | MH208628.1 | China |  |
|  |  |  |  |  | PQ001987.1 | Pakistan |  |
|  |  |  |  |  | LC326009.2 | Myanmar |  |
|  |  |  |  |  | PP702140.1 | India |  |
| **7** | *18S rRNA* | PV269853 | *Th. uilenbergi* | 98.17–99.77% | MW881299.1 | China |  |
|  |  |  |  |  | KF781308.1 | Egypt |  |
|  |  |  |  |  | MN544922.1 | Iraq |  |
| **8** | *gltA* | PV335567 | *Rickettsia (R) slovaca* | 100% | CP002428.1 | France | Figure 5 A,B |
|  |  |  |  |  | U59725.1 |  |  |
|  |  |  |  |  | KX506730.1 | China |  |
|  |  |  |  |  | MT293345.1 | Iran |  |
|  |  |  |  |  | CP003375.1 | USA |  |
| **9** | *gltA* | PV335568 | *R. sibirica* | 99.88–100% | CP170612.1 | China |  |
|  |  |  |  |  | KU310587.1 | Russia |  |
|  |  |  |  |  | U59734.1 | France |  |
|  |  |  |  |  | MT293349.1 | Iran |  |
| **10** | *Sca4* | PV335570 | *R. slovaca* | 100% | MW430426.1 | Kazakhstan |  |
|  |  |  |  |  | CP002428.1 | France |  |
|  |  |  |  |  | OR148338.1 | Russia |  |
| **11** | *Sca4* | PV335569 | *R. sibirica* | 99.43–100% | CP170612.1 | China |  |
|  |  |  |  |  | HM050295.1 | Senegal |  |
| **12** | *Sca4* | PV339643 | *R. conorii* subsp. raoultii | 99.55–99.77% | OR148327.1 | Russia |  |
|  |  |  |  |  | CP098324.1 | China |  |
| **13** | *ompA* | PV364127 | *R. slovaca* | 100% | CP002428.1 | France | Figure 6 A,B |
|  |  |  |  |  | MF379303.1 | Turkey |  |
|  |  |  |  |  | MW779483.1 | Iran |  |
|  |  |  |  |  | CP003375.1 | USA |  |
|  |  |  |  |  | KX506733.1 | China |  |
| **14** | *ompA* | PV364128 | *R. sibirica* | 100% | U43807.1 | France |  |
|  |  |  |  |  | CP170612.1 | China |  |
| **15** | *ompA* | PV364129 | *R. raoultii* | 100% | MH932055.1 | China |  |
|  |  |  |  |  | MK304548.1 | Russia |  |
|  |  |  |  |  | CP010969.1 | France |  |
| **16** | *ompA* | PV364130 | *R. conorii* subsp. raoultii | 100% | HM161789.1 | Italy |  |
|  |  |  |  |  | PP465040.1 | Turkey |  |
|  |  |  |  |  | KX506737.1 | China |  |
| **17** | *ompB* | PV364131 | *R. slovaca* | 100% | MW430423.1 | Kazakhstan |  |
|  |  |  |  |  | CP002428.1 | Slovakia |  |
|  |  |  |  |  | AF123723.2 |  |  |
|  |  |  |  |  | MH532273.1 | Italy |  |
|  |  |  |  |  | MT557701.1 | Russia |  |
|  |  |  |  |  | CP003375.1 | USA |  |
| **18** | *ompB* | PV364132 | *R. sibirica* | 99.39–99.75% | PP835457.1 | Morocco |  |
|  |  |  |  |  | AF123722.1 | France |  |
|  |  |  |  |  | CP170614.1 | China |  |
